# Mediterranean Long Shelf-Life Landraces: An Untapped Genetic Resource for Tomato Improvement

**DOI:** 10.3389/fpls.2019.01651

**Published:** 2020-01-10

**Authors:** Miquel À. Conesa, Mateu Fullana-Pericàs, Antonio Granell, Jeroni Galmés

**Affiliations:** ^1^ Department Biologia-INAGEA, Universitat de les Illes Balears, Balearic Islands, Spain; ^2^ Instituto de Biología Molecular y Celular de Plantas, CSIC-UPV, Valencia, Spain

**Keywords:** drought tolerance, extended fruit shelf-life, fruit quality traits, gas exchange, Mediterranean landraces, tomato, yield

## Abstract

The Mediterranean long shelf-life (LSL) tomatoes are a group of landraces with a fruit remaining sound up to 6–12 months after harvest. Most have been selected under semi-arid Mediterranean summer conditions with poor irrigation or rain-fed and thus, are drought tolerant. Besides the convergence in the latter traits, local selection criteria have been very variable, leading to a wide variation in fruit morphology and quality traits. The different soil characteristics and agricultural management techniques across the Mediterranean denote also a wide range of plant adaptive traits to different conditions. Despite the notorious traits for fruit quality and environment adaptation, the LSL landraces have been poorly exploited in tomato breeding programs, which rely basically on wild tomato species. In this review, we describe most of the information currently available for Mediterranean LSL landraces in order to highlight the importance of this genetic resource. We focus on the origin and diversity, the main selective traits, and the determinants of the extended fruit shelf-life and the drought tolerance. Altogether, the Mediterranean LSL landraces are a very valuable heritage to be revalued, since constitutes an alternative source to improve fruit quality and shelf-life in tomato, and to breed for more resilient cultivars under the predicted climate change conditions.

## Introduction

The Mediterranean “long shelf-life” (LSL) or “long storage” tomatoes (*Solanum lycopersicum* L.) are a group of landraces that converge in three characteristic traits. First, the LSL fruit phenotype, allowing tomato fruits to remain sound, without symptoms of wrinkling or deterioration, for up to 6-12 months after harvest (e.g., Casals et al., 2011; Bota et al., 2014; Manzo et al., 2018; Tranchida-Lombardo et al., 2018a). In contrast with the commercial practice for most tomato cultivars, LSL fruits are harvested fully ripen on the vine, needing neither postharvest ripening nor cold temperature storage, which maximizes fruit quality. Second, landraces are drought tolerant as a result of typical open field cultivation under semi-arid Mediterranean summer conditions, meaning that plants are irrigated only during first stages after transplantation and rely on occasional rain-fed afterwards. This has been an unconscious selection (Zohary, 2004; Meyer et al., 2012) imposed by local conditions and the efforts of local farmers to cultivate tomato during last centuries in the Mediterranean basin (e.g., Pernice et al., 2010; Galmés et al., 2011; Galmés et al., 2013; Mercati et al., 2015; Patanè et al., 2016; Siracusa et al., 2018). Besides the convergence in extended shelf-life and drought tolerance, the third defining trait is the huge variation in fruit morphology and quality traits, plant habit and agronomic performance within and among landraces (Ochogavía et al., 2011; Bota et al., 2014). This trait responds to different selection criteria across regions, including tomato cultivation practices, soil characteristics, cultural practices, culinary uses and preferences in local populations (Zeven, 1998; Camacho-Villa et al., 2005).

In this study, we review most of the available information characterizing the Mediterranean LSL landraces, to focus on the origin, distribution and diversity, the underlying causes of the extended shelf-life, drought tolerance and variation in fruit morphology and quality traits, and the potentiality of this important genetic resource for future tomato improvement. Apart of a reservoir of useful alleles, Mediterranean LSL landraces also represent a fascinating treasure of culture and heritage, stories and possibilities of ancient tomato cultivation in the Mediterranean area.

## Origin, Diversity and Distribution of the LSL Landraces

After its introduction in Europe by the 16^th^ century, the tomato suffered a rapid diversification (Sims, 1980; Ruiz et al., 2005; Rodriguez et al., 2011). Tomato was initially used as ornamental, what probably promoted increased variation in traits like fruit colour and shape. This is evidenced in pictograms from the second half of the 16^th^ century, frequently representing chimeric plants mirroring the existing diversity (e.g., Fuchs, ca. 1550; Matthiolus, 1586; see Daunay et al., 2007, and Peralta et al., 2008). Diversification was particularly important in the Mediterranean basin, which is considered a secondary centre of diversity of the species (Esquinas-Alcázar and Nuez, 1995; Cebolla-Cornejo et al., 2007; Mazzucato et al., 2008; Terzopoulos and Bebeli, 2010; García-Martínez et al., 2013). Despite the exact introduction of tomato in the Mediterranean basin is unknown, in the Balearic Islands several paintings dated ca. 1630–1650 show leaves and fruits similar to the ‘de Ramellet’ landrace (Sa Nostra, 1994), denoting that selection for this typical landrace could extend back for four centuries in this region.

Genetic studies in tomato suggest notoriously different selection related to geographic differences and regional preferences (Sim et al., 2011; Sim et al., 2012; Blanca et al., 2015). Accordingly, selection for fruit colour, size and shape varied depending on the region which, together with low levels of out-crossing typical in tomato crop, led to a large number of family- or local-inherited varieties (Watson, 1996; Zeven, 1998), and the maintenance of very local landraces associated to regional uses and culture in the Mediterranean basin.

Yield and fruit size have been in general the main drivers for tomato selection (Bai and Lindhout, 2007; Rodriguez et al., 2011; Lin et al., 2014). Instead, in the Mediterranean basin, the extended shelf-life of fruits has been a determining selection factor, together with plant drought tolerance. Consequently, traits characterizing most non-LSL landraces, like fruit shape, size and color, are usually very variable in the Mediterranean LSL landraces.

### Diversity and Distribution of the Mediterranean LSL Landraces

Noteworthy, the largest array of LSL landraces exists in the Eastern Iberian Peninsula, the Balearic Islands, southern Italy and Sicily; all these regions forming part of the Crown of Aragón from the end of 13^th^ to early 18^th^ centuries (Bisson, 1986). This could have been a factor promoting the expansion of particular landraces, cultural practices and tomato uses across the kingdom, and was suggested as a reason explaining the lack of country-specific population structure when comparing non-LSL traditional accessions from Spain and Italy (García-Martínez et al., 2013). In the following, we summarize the occurrence of Mediterranean LSL landraces for which information exists in literature. Those landraces have been compiled in **Table 1**.

**Table 1:** Mediterranean long shelf-life (LSL) landraces ordinated by regions of origin. The published names are indicated, together with some general details for common shelf-life (d. are days and m. are months) and fruit size and shape as described in the literature.

Landrace	Region	Shelf-life	Fruit size	Fruit shape	References
de Ramellet	Balearic Islands	6–12 m.; 49% fruits after 6 m.	47–57 g (see **Tables 3** and **4**)	round-slightly flat (but see **Figure 1**)	1–6
de Penjar	Catalonia/Valencian Comm.	127 d. (78–139 d.)	38–86 g (25–121 g)	round (but variable)	7,8
Vesuviano (Piennolo Vesuviano; Pomodorino del Piennolo del Vesuvio)	Campania–Vesuvius area	50% fruits after 6 m.	20–35 g	oval; apex	9–16
ecotype Fiaschella	Campania–Vesuvius area				11,13,14
ecotype Lampadina	Campania–Vesuvius area				11,13,14
ecotype Patanara	Campania–Vesuvius area				11,13,14
ecotype Re Umberto	Campania–Vesuvius area				11,13,14
Acampora	Campania–Vesuvius area				16
Lucariello	Campania–Vesuvius area	5–10 m.; (56% fruits after 60 d.)		heart-shape; apex	17,16,18
Pomodoro di Ercolano	Campania–Vesuvius area		ca. 26 g		15,16
Piennolo Giallo	Campania–Napoli–Visciano				16
Giallo Beneventano	Campania–Benevento	(very long)			17,16
Castel di Sasso	Campania–Caserta				16
Seccagno 1	Campania–Avellino				16
Corbarino (Pomodorino di Corbara)	Campania–Salerno	37% fruits after 60 d.	15–21 g; > 21 g	round/oval-pear/elongate; variable apex	19,13,20
Nocerino	Campania–Salerno–Nocera		12–21 g	round/oval-pear/elongate; variable apex	19,9,16
Casarbore	Campania				16
Crovarese (Corbarino type)	Campania (commercial)	(long)			17,21,16,18
Principe Borghese	Campania (commercial)		15–18 g	round/oval/elongate; apex	19,22,23,9,11, 24,13,14,15,16
Regina	Apulia		22 g		9,10,25,16
ecotype di Fasano	Apulia –Fasano		22 g		25
ecotype di Monopoli	Apulia –Monopoli		22 g		25
ecotype di Ostuni	Apulia –Ostuni		22 g		9,10,25,16
Locale di Altamura	Apulia –Bari		ca. 22 g		15,16
Pummidora Scimona (Pomodoro da Serbo Giallo)	Apulia–Grecìa				26
Locale di Arnesano	Apulia –Lecce		ca. 39 g		15,16
Giallo di Pitigliano	Tuscany	(several months)			27
Perina a Punta della Valtiberina	Tuscany	(up to next spring)			(link in 27)
Rosso di Pitigliano (da Serbo Rosso)	Tuscany				27
Tondino Liscio da Serbo Toscano (Liscio da Serbo; Pomodorino da Appendere; Tondino Maremmano)	Tuscany	(for winter)			27
Buttigghieddu d'appenniri	Sicily–Agrigento		ca. 17 g		9,10,15,16
Kachi di Sciacca	Sicily–Agrigento		ca. 31 g		15,16
Linosa	Sicily–Agrigento				9,10,16
Mezzocachi di Montallegro	Sicily–Agrigento		ca. 37 g		15,16
Piriddu (Piruddu)	Sicily–Agrigento–Siculana				9,10,16
Pizzottello di Montallegro	Sicily–Agrigento		18–21 g (ca. 27 g)	round; apex	22,23,24,30,15,16
Pizzutello di Licata	Sicily–Agrigento		ca. 21 g		15,16
Pizzutello di Montallegro	Sicily–Agrigento		ca. 27 g		15,16
Pizzutello di Sciacca	Sicily–Agrigento		< 12 g	elongate; apex	22,24,28,15,16
San Andrea (di Lampedusa)	Sicily–Agrigento				9,16
Albicocca di Lipari	Sicily–Messina		ca. 33 g		29
Locale di Basicò Giallo	Sicily–Messina		18–21 g (ca. 27 g)	round; apex	22,24,15,16
Locale di Basicò Rosso	Sicily–Messina		ca. 24 g		15,16
Locale di Filicudi	Sicily–Messina		12–15 g (ca. 20 g)	round	22,23,24,30,15,16
Locale di Pollara (Locale di Salina 9)	Sicily–Messina		< 12 g (11–21 g)	round	22,24,15,16
Locale di Salina 1	Sicily–Messina		ca. 21 g		15,16
Locale di Salina 2	Sicily–Messina		15–18 g	round; apex	22,24,15,16
Locale di Salina 3	Sicily–Messina		ca. 42 g		15,16
Locale di Salina 4	Sicily–Messina		ca. 24 g		15,16
Locale di Salina 5	Sicily–Messina		ca. 48 g		15,16
Locale di Salina 6	Sicily–Messina		12–15 g	round	22,24,12,28,15,16
Locale di Salina 10	Sicily–Messina		ca. 26 g		15,16
Locale di Vulcano	Sicily–Messina		ca. 17 g		15,16
Mazzarrà San Andrea	Sicily–Messina		ca. 23 g		15,16
Ruccaloru	Sicily–Messina –S.Pierniceto		12–15 g (ca. 25 g)	elongate	22,24,15,16
Poma	Sicily–Palermo–Cefalù				9,16
Albicocca di Favignana	Sicily–Trapani–Egadi Is.		ca. 25 g		29
Locale di Custonaci	Sicily–Trapani		18–21 g (ca. 28 g)	round; apex	22,24,15,16
Paceco	Sicily–Trapani				9
Patataro	Sicily–Trapani–Marsala				9,10,16
Pizzutello d'Inverno	Sicily–Trapani–Marsala				9,10,16
Pizzutello di Nubia	Sicily–Trapani				9,10,16
Pizzutello di Paceco	Sicily–Trapani				10,16
Sinacori	Sicily–Trapani–Paceco				9,10,16
Giallo Piccolo a Punta	Sicily		ca. 31 g		29
*Landrace Siciliana*	Sicily	(long)			17
Percopara	Sicily		ca. 41 g		29
Pizzutello (di Stagnone)?	Sicily–Trapani		ca. 25 g		29
Rosso	Sicily		ca. 24 g		29
Stella	Sicily(Palermo)				9,10

The references for the information are indicated: 1-Galmés et al., 2011; 2-Ochogavía et al., 2011; 3-Galmés et al., 2013; 4-Bota et al., 2014; 5-Fullana-Pericàs et al., 2017; 6-Fullana-Pericàs et al., 2019; 7-Casals et al., 2012; 8-Figàs et al., 2015; 9-Mercati et al., 2015; 10-Abenavoli et al., 2016; 11-Fattore et al., 2016; 12-Guida et al., 2017; 13-Sacco et al., 2017; 14-Manzo et al., 2018; 15-Siracusa et al., 2018; 16-Tranchida-Lombardo et al., 2018b; 17-Landi et al., 2017; 18-Tranchida-Lombardo et al., 2018a; 19-Andreakis et al., 2004; 20-Pernice et al., 2010; 21-Tamburino et al., 2017; 22-Siracusa et al., 2012; 23-Siracusa et al., 2013; 24-Patanè et al., 2016; 25-Renna et al., 2018; 26-Laghetti et al., 2008; 27-Berni et al., 2018; 28-Giorio et al., 2018; 29-Barbagallo et al., 2008; 30-Patanè et al., 2017.

**Figure 1:**
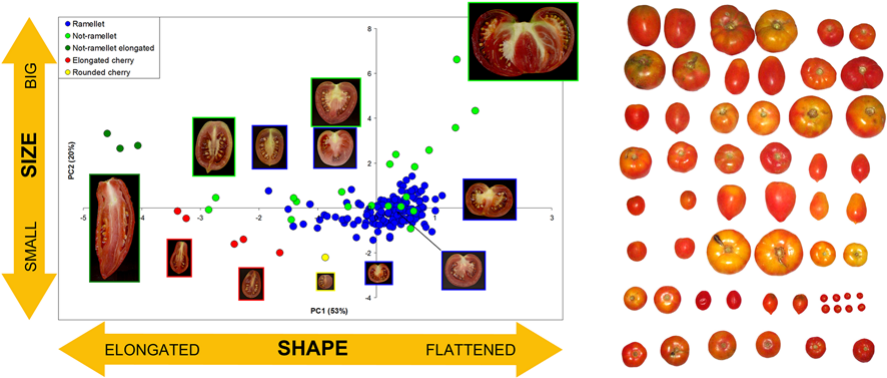
Morphological variation in fruit size and shape in the Balearic LSL landrace ‘de Ramellet.’ Left: ‘de Ramellet’ fruit (blue dots) as compared to diverse tomatoes with variable size and shape, based on the first two Principle Components (73% of total variation explained) resulting from morphological data collected from transverse sections with Tomato Analyzer (Brewer et al., 2006). Each accession is represented by an average of all scanned fruits. Representative fruits are shown along the plot. PC1 and PC2 mainly explain fruit size and fruit elongation, respectively. Modified from Conesa et al. (2010). Right: Variation found in a prospection across the Balearic Islands to create the UIB-collection. Modified from Ochogavía et al. (2011).

The ‘de Ramellet’ tomato from the Balearic Islands is a landrace, or population of landraces (Zeven, 1998; Camacho-Villa et al., 2005) consisting of diverse inbreeding lines maintained in family orchards, small farms and cooperatives, used for self-consumption and sold in small markets, cultivated following traditional management practices, and with seed being self-stored and maintained within families over decades. This selection and seed storage system, and the lack of a single morphological type preferred by the Balearic people, resulted in an enormous heterogeneity in fruit morphology and size due to variable preferences among growers (**Figure 1**), as documented through a prospection for variation across the Balearic Islands (Conesa et al., 2010; Ochogavía et al., 2011; Bota et al., 2014). Results denoted that some growers maintain different landraces based on the particular fruit properties and uses they consider interesting, e.g., a flat-fruit landrace for fresh consumption (rubbed on bread), and an elongated-fruit landrace for cooking and canning after pre-cooking, which is another factor boosting variation within this landrace. Balearics recognize ‘de Ramellet’ as a particularly acid tomato, unsuitable to consume in salad. It is used for cooking, but the most generalized use is to rub on bread. In this regard, consumers appreciate that the skin, which is thick and hard, remains completely free of pericarp after spreading, which is uncommon in diverse Iberian Peninsula LSL landraces, and especially in commercialized ‘de Ramellet’-like F_1_ hybrids. Some landraces have proven to be tolerant to pests (Ximénez-Embún et al., 2018).

The ‘de Penjar’ tomato from Catalonia and Valencia has similar fruit morphology and cultivation practices than ‘de Ramellet’ (Casals et al., 2011). The close vicinity and historical and cultural links with the Balearic Islands presupposed a close relationship between both landraces, although morphological and physiological variation in ‘de Ramellet’ seems to be wider (Bota et al., 2014; Fullana-Pericàs et al., 2017; Fullana-Pericàs et al., 2019) and genetic variation denotes particular identity for the Balearic accessions (A. Granell, unpubl.).

Various ‘de Ramellet’-like and ‘de Penjar’-like F_1_ hybrids are widely produced by professional growers, and some seed companies produce different ‘de Ramellet’-like and ‘de Penjar’-like varieties improved with biotic tolerances and uniform color. The original landrace material for such breeding is in many cases uncertain and product traceability suggests that in some cases the same F_1_ hybrid is sold either as ‘de Penjar’ or ‘de Ramellet’ depending on the market (i.e., Catalonia-Valencia or Balearic Islands, respectively).

LSL accessions similar to ‘de Ramellet’ and ‘de Penjar,’ with poorly defined and little extended use, and mainly of unknown origin, are found in most part of the Mediterranean coast of the Iberian Peninsula, and also inland regions like Andalusia and Cáceres (R. Fernández-Muñoz, pers. comm.). The latter location is only ca. 250 km away from Alcobaça in Portugal, location from which the ‘Alcobaça’ landrace was described (Almeida, 1961; Kopeliovitch et al., 1980). This could indicate that LSL landraces anciently existed in the Iberian Peninsula aside the Mediterranean coast. Most LSL accessions occurring outside Catalan-speaking regions are recognized as ‘Alcobaça,’ textually meaning ‘de Penjar’ (“for hanging”) in Spanish. In fact, some commercial tomatoes are recognized as ‘Alcobaça’ also in Algeria (Maamar et al., 2015).

Attending to literature, Sicily is by far the richest Italian region for LSL landraces, with outstanding groups of landraces under names like ‘Pizzutello’ and ‘Locale’ followed by the name of the region of provenance (**Table 1**). Those are in general cultivated in very small family orchards, mostly for self-consumption (Sacco et al., 2017). This results in relatively low productive landraces as compared to commercial cultivars (Siracusa et al., 2013). Fruits are small, in many cases below 25 g. Fruit shape is variable, from round to elongated or pear-shaped, frequently with a prominent stylar end tip, which is variable or absent across landraces (**Table 1**). Despite the extended shelf-life might have been the primary selection criteria, most have high to very high sugar content (**Table 2**), denoting that this has also been an important selective trait. Drought tolerance is common in Sicilian landraces because anciently, but still today, have been cultivated under rain-fed conditions. To some extent, this also responds to the increased fruit quality obtained under such conditions (e.g., Siracusa et al., 2012; Siracusa et al., 2013; Sacco et al., 2017).

**Table 2:** Agronomic and fruit quality traits in Mediterranean long shelf-life (LSL) landraces, ordinated by regions of origin. For landrace details, see **Table 1**. The number of accessions in the average (n) is indicated unless is one. Cultivation conditions and different water treatments indicated when available: OF, open field in Mediterranean summer; GH, greenhouse; WW, full irrigation replacing potential evapotranspiration (PET); WD, water deficit (number in brackets indicate the % of covered PET); and RF, rain-fed. Yield and fruit number are per plant unless units are indicated. Total soluble solids, titratable acidity and firmness are in °Brix, g citric ac. 100 g^−1^ and shore units, respectively, unless units are indicated. When different water treatments were applied, data is indicated in the same order as treatments and separated by semicolon. Asterisk (*) indicates significant differences between treatments, when available. For any trait, if a range was indicated for different accessions, it is shown after the average in brackets. Fruit quality traits measured at harvest time unless months (m.) or days (d) indicated in brackets.

Landrace	Region	Cultivation	Yield (kg pl^−1^)	Fruit num.	Fruit weight (g)	°Brix	Titratable ac. (g citric ac. 100g^−1^)	pH	Firmness	Refs.
Ramellet (n = 6)	Balearic Islands	OF–WW; WD(20%PET)	4.1; 1.3*	76; 60*	57; 23*					1,2
Ramellet (n = 158)	Balearic Islands	OF–WW	1.3 (0.1–2.8)	29 (4–75)	47 (9–164)	5.9(0d)–(170d)5.1*	1.48(0d)–(170d)0.71*	3.85(0d)–(170d)4.49*	1.14(0d)–(170d)1.56*	3,4
Ramellet (n = 48)	Balearic Islands	OF–WW; WD(40%PET)	2.6; 2.1*	89; 74*	30; 28	4.8; 5.7	1.09; 1.15*	4.25; 4.31*		5
Ramellet (n = 4)	Balearic Islands	OF; GH	4.2; 2.8		105; 76	6.1; 5.9	0.58; 0.53	4.15; 4.14	53.1; 50.2	6
Penjar (n = 4)	Catalonia/Val. Comm.	OF–WW	3.0 (1.7–3.1)		67 (31–116)	5.3 (4.8–6.6)				7
Penjar (n = 27)	Cat./Val Comm./Bal. Is.	OF–WW	2.9 (1.6–4.3)		64 (25–121)	6.9 (5.0–9.5)				8
Penjar (n = 2agro; n = 5qualit)	Valencian Comm.	OF–WW	2.5 (0.3–6.5)		200 (183–217)	4.7 (4.1–5.4)	0.56 (0.39–0.78)	4.3 (4.1–4.5)		9
Penjar (n = 3)	Valencian Comm.	WW–OF; GH	3.7; 2.4		70; 51	6.6; 7.2	0.42; 0.45	4.37; 4.33	60.9; 45.3	6
Penjar (n = 12)	Valencian Comm.	OF–WW			(36–86)	6.6 (4.1–8.7)	0.54 (0.35–0.81)	4.25 (3.98–4.45)		10
PenjarVAL (n = 6)	Valencian Comm.	OF–WW; WD(40%PET)	2.3; 1.5*	89; 79	35; 23	4.4; 5.6	0.84; 1.08*	4.30; 4.25		5
PenjarCAT (n = 6)	Catalonia	OF–WW; WD(40%PET)	1.7; 1.7	96; 86	15; 18*	5.7; 5.8	1.30; 0.92	4.24; 4.31		5
DFD	Catalonia	GH–WW				5.5				11
Ramellet/Penjar improved (n = 3)	Commercial improved	WW–OF; GH	3.7; 3.5		97; 64	6.1; 6.5	0.45; 0.51	4.31; 4.3	53.9; 47.9	6
Ramellet/Penjar hybrids F1 (n = 2)	Commercial F1	WW–OF; GH	4.4; 3.4		100; 76	6.5; 6.6	0.49; 0.54	4.25; 4.16	59.8; 58.3	6
Piennolo del Vesuvio	Campania	OF–WW; RF (wet year)	5.3; 5.1	160; 183	32.8; 27.5*	4.7; 6.2*		4.45; 4.56		12
Vesuvio	Campania–Ercolano	OF–RF			25	7.5	0.34			13
Piennolo Vesuviano	Campania–Ercolano	N/A (field collected)				6.9(0m.)–(6m.)7.9*	0.53(0m.)–(6m.)0.50	4.36(0m.)–(6m.)4.45		14
Lucariello	Campania	OF–RF				8.0				15
Ercolano	Campania–Ercolano	OF–RF			26	7.7	0.35			13
Corbarino	Campania	OF–RF				7.2				15
Corbarino (n = 4)	Campania–Sarno Valley	OF–4m. irrig.(April–Sept)	73.9 t/ha		18	6.3	0.4	4.5		16
Corbarino PC01	Campania	OF–WW; WD(50%PET); RF	114; 98; 62 t/ha		17; 17; 13	5.5; 5.6; 7.3				17
Corbarino PC05	Campania	OF–WW; WD(50%PET); RF	122; 112; 72 t/ha		17; 17; 13	6.3; 6.8; 9.1				17
Principe Borghese	Campania (commercial)	OF–RF			17	7.4	0.32			13
Principe Borghese	Campania (commercial)	OF–RF	13.7 t/ha							18
Principe Borghese	Campania (commercial)	OF–RF	16.8 t/ha		15	7.6	0.31			19
Regina–Fasano ecotype	Puglia–Fasano	OF– RF			20	6.4c				20
Regina–Monopoli ecotype	Puglia–Monopoli	OF– RF			22	7.0b				20
Regina–Ostuni ecotype	Puglia–Ostuni	OF–RF			25	7.6				20
Altamura	Puglia–Bari	OF– RF			22	6.9	0.34			13
Arnesano	Puglia–Lecce	OF– RF			39	7.7	0.33			13
Buttigghieddu	Sicily–Agrigento	OF– RF			17	7.4	0.31			13
Kachi di Sciacca	Sicily–Agrigento	OF– RF			31	6.8	0.32			13
Mezzocachi di Montallegro	Sicily–Agrigento	OF– RF			37	6.2	0.28			13
Pizzottello di Montallegro	Sicily–Agrigento	OF–RF	(18.5–19.8 t/ha)		(19–27)	(7.0–8.0)	0.29	4.07	22.7	21,19,18,22,13
Pizzutello di Licata	Sicily–Agrigento	OF–RF			(21–22)	(6.2–6.7)	0.34			13
Pizzutello di Montallegro	Sicily–Agrigento	OF– RF			22	6.2	0.32			13
Pizzutello di Sciacca	Sicily–Agrigento	OF– RF	13.6 t/ha		12	8.1	0.38			18,13
Albicocca di Lipari	Sicily–Messina	GH–WW; WD(50%PET)			33; 16*	5.5; 6.6*	2.09; 2.41	4.25; 4.19		23
Locale di Basicò Giallo	Sicily–Messina	OF– RF	11.7 t/ha		27	6.5	0.26			18,13
Locale di Basicò Rosso	Sicily–Messina	OF– RF			24	6.7	0.3			13
Locale di Filicudi	Sicily–Messina	OF– RF	(20.8–23.4 t/ha)		14	8.2	0.34	4.08	20.4	19,18,22
Locale di Filicudi	Sicily–Messina	OF– RF			20	7	0.36			13
Locale di Pollara	Sicily–Messina	OF– RF	18.2 t/ha		12	7.6	0.29			18,13
Locale di Salina 1	Sicily–Messina	OF– RF			21	6.8	0.34			13
Locale di Salina 2	Sicily–Messina	OF– RF	28.9 t/ha		22	7.5	0.34			18,13
Locale di Salina 3	Sicily–Messina	OF– RF			42	6.6	0.34			13
Locale di Salina 4	Sicily–Messina	OF– RF			24	7.4	0.32			13
Locale di Salina 5	Sicily–Messina	OF– RF			48	5.6	0.27			13
Locale di Salina 6	Sicily–Messina	OF– RF	16.1 t/ha		15	7.4	0.33			18,13
Locale di Salina 6	Sicily–Messina	OF–WW; RF (dryY)	3.2; 3.1	306; 283	11; 11	4.9; 6.0*				12
Locale di Salina 6	Sicily–Messina	OF–WW; rainfed(wetY)	3.4; 3.2	251; 238	14; 14	5.5; 7.5*		4.39; 4.57		12
Locale di Salina 10	Sicily–Messina	OF– RF			26	7.3	0.31			13
Locale di Vulcano	Sicily–Messina	OF– RF			17	7.5	0.26			13
Mazzarrà S. Andrea	Sicily–Messina	OF– RF			23	7.3	0.28			13
Ruccaloru	Sicily–Messina –S.Pierniceto	OF– RF	13.2 t/ha		25	7.2	0.28			18,13
Albicocca di Favignana	Sicily–Trapani (Egadi Is.)	GH–WW; WD(50%PET)			25; 24	6.0; 7.8*	1.86; 2.75*	4.02; 4.22		23
Locale di Custonaci	Sicily–Trapani	OF– RF	22 t/ha		28	7.3	0.29			18,13
Giallo Piccolo a Punta	Sicily	GH– WW; WD(50%PET)			31; 26*	5.5; 7.3*	1.63; 2.75*	4.21; 4.11		23
Percopara	Sicily	GH– WW; WD(50%PET)			41; 23*	6.0; 6.8*	1.69; 2.28*	4.30; 4.27		23
Pizzutello di Stagnone	Sicily–Trapani	GH– WW; WD(50%PET)			27; 24	5.8; 6.8*	1.93; 2.24	4.27; 4.23		23
Rosso	Sicily	GH– WW; WD(50%PET)			24; 16*	5.9; 6.3	1.80; 2.80*	4.10; 3.94		23

The references for the information are indicated: 1-Galmés et al., 2011; 2-Galmés et al., 2013; 3-Ochogavía et al., 2011; 4-Bota et al., 2014; 5-Fullana-Pericàs et al., 2019; 6-Figàs et al., 2018; 7-Casals et al., 2011; 8-Casals et al., 2012; 9-Cebolla-Cornejo et al., 2013; 10-Figàs et al., 2015; 11-Saladie et al., 2007; 12-Guida et al., 2017; 13-Siracusa et al., 2018; 14-Manzo et al., 2018; 15-Tranchida-Lombardo et al., 2018a; 16- Andreakis et al., 2004; 17-Pernice et al., 2010; 18-Patanè et al., 2016; 19-Siracusa et al., 2013; 20-Renna et al., 2018; 21-Siracusa et al., 2012; 22-Patanè et al., 2017; 23-Barbagallo et al., 2008.

Diverse landraces from mount Vesuvio region in Campania are grouped into the ‘Vesuviano’ landrace, which is cultivated since the end of the 19^th^ century (Ercolano et al., 2014). Nevertheless, different ecotypes are also recognized, like ‘Fiaschella,’ ‘Lampadina,’ ‘Patanara,’ ‘Re Umberto’ and ‘Principe Borghese’ (**Table 1**), all included in the Protected Designation of Origin regulation (PDO) ‘Pomodorino del Piennolo del Vesuvio.’ Tomatoes under this PDO may have a minimum of 6.5 °Brix, which denotes that sugar content has been an important selective trait (Manzo et al., 2018).

Some other renowned LSL landraces in Campania are ‘Lucariello,’ which is a ‘Vesuviano’-like tomato with shelf-life of 5–10 months (Tranchida-Lombardo et al., 2018a), and ‘Corbarino.’ The latter is used either fresh, canned or to store hung, with notorious diversity in fruit shape among landraces (**Table 1**). Most ‘Corbarino’ landraces are very vigorous, outperforming commercial F_1_ hybrids in yield and quality traits (Andreakis et al., 2004; Sinesio et al., 2007; Lisanti et al., 2008), and are particularly drought tolerant (Pernice et al., 2010).

The ‘Regina’ tomato includes diverse drought-adapted LSL landraces in Puglia, with average shelf-life of ca. 6 months (Renna et al., 2018). Also, some LSL landraces in Tuscany are ‘Tondino Liscio da Serbo Toscano,’ ‘Giallo di Pitigliano,’ ‘Rosso di Pitigliano,’ and ‘Perina a Punta della Valtiberina’ (Berni et al., 2018), from which little has been published. This might be extensible to further regions in Italy.

LSL landraces might also be found in other Mediterranean regions, but little information has been reported to our knowledge. For example, landraces in Greece have been tested for genetic, morphological and agronomic traits (Terzopoulos and Bebeli, 2008; Terzopoulos et al., 2009; Terzopoulos and Bebeli, 2010; Koutsika-Sotiriou et al., 2016), showing that local selection favoured plant morphology and fruit shape and flavour over shelf-life (Terzopoulos and Bebeli, 2010), which would hinder for the existence of LSL landraces. Landraces of supposed Greek origin have been documented in Greek-speaking regions in Southern Italy (Grecìa), like ‘pummidora scimona,’ (‘pomodoro da serbo giallo’ in Italian), a yellow-colored LSL landrace (Laghetti et al., 2008).

## The Extended Shelf-Life in Mediterranean LSL Landraces

### Selection for the LSL Fruit Phenotype

A clear indication that extended shelf-life was the main selective trait is indeed the local name of these landraces. Thus, names for ‘de Ramellet,’ ‘de Penjar,’ ‘Alcobaça’ in the Balearic Islands and Iberian Peninsula, and ‘da Serbo,’ ‘da Appendere,’ ‘del Piennolo,’ ‘d'Inverno,’ etc. in Italian landraces, all refer to the use of tomatoes for long-term-over-winter-storage, disposed in bunches, or to be hung (e.g., Casals et al., 2011; Bota et al., 2014; Mercati et al., 2015; Manzo et al., 2018).

**Figure 2:**
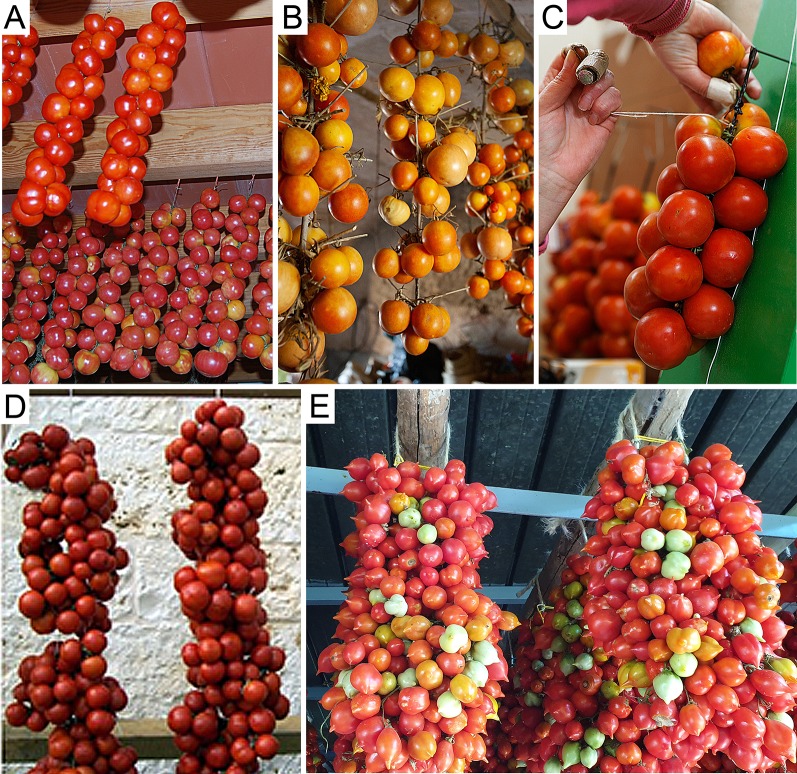
Different ways to store hung long shelf-life tomatoes across the Mediterranean. **(A)** Typical ‘de Ramellet’ strings with fruit pedicels needle-sewn to a main rope (Banyalbufar, Mallorca; courtesy: Aina Socies - Associació de Varietats Locals de Mallorca). **(B)** ‘de Ramellet’ hung by pedicels on wild olive tree branches (Artà, Mallorca; courtesy: Toni Muñoz). **(C)** Needle-sewing of ‘de Penjar’ strings (Alcalà de Xivert, Castelló; courtesy: Associació de Productors i Comercialitzadors de Tomata de Penjar d'Alcalà de Xivert). **(D)** Typical Sicilian ‘da Serbo’ trusses (Sicily). **(E)** Typical ‘piennoli’ of ‘Pomodorino del Piennolo del Vesuvio’ (Ercolano, Napoli; courtesy: Rosario Custro).

In the Balearic Islands, the ‘de Ramellet’ name (‘small-bunch’ in Catalan) may refer to the fruit bunch in the plant, or perhaps to the strings anciently produced to store fruits. Thus, an ancient practice still performed to date consists in sewing the tomato pedicels to a main rope, threading fruit strings of variable length, anciently hung on the roof beams in ventilated sheds (**Figure 2**). This was industrialized ca. 1930 in Banyalbufar, a village of Mallorca that build up an industry and exported ‘de Ramellet’ mainly to Catalonia (Fairchild, 1927). This could be an important route for genetic material exchange between Mallorca and Catalonia. In fact, the ‘de Penjar’ tomatoes from Catalonia and Valencia are stored in very similar bunches, also sewing the pedicels to a main rope (**Figure 2**). It is worth mentioning that other traditional ways to preserve ‘de Ramellet’ exist in the Balearic Islands, like hanging tomatoes in very densely branched olive tree woods through the fruit bunch stems (**Figure 2**), or disposing individual fruits onto hurdle to ensure ventilation.

Among Italian landraces, the individual fruit threading performed in ‘de Ramellet’ and ‘de Penjar’ is not common. It seems to be performed in the ‘Regina’ landrace from Puglia, by tying fruit pedicels with a cotton thread in bunches called ‘ramasole’ (Sacco et al., 2017; Renna et al., 2018). Fruit hanging in most Italian “da Serbo” landraces is performed without detaching fruits from the truss, and tying together different trusses twisted arround a twine, sometimes conforming a circle (**Figure 2**), (Mercati et al., 2015; Sacco et al., 2017; Manzo et al., 2018). This is also common in the ‘Vesuviano’ landraces from Campania, whose bunches are called ‘piennolo’ or ‘spunzilli’ (**Figure 2**), (Sacco et al., 2017).

### Genetic Basis of the LSL Phenotype

The tomato ripening mutants like *rin*, *nor*, *Nr, Gr,* and *Cnr*, among others, result in a strong ripening impairment frequently related to ethylene insensitivity (Barry and Giovannoni, 2007; Giovannoni, 2007), and with pleiotropic effects on colour and flavour. Because of that, *rin* and *nor* are used only in heterozygous in breeding programs, resulting in delayed and altered ripening and extended shelf-life (Garg et al., 2008; Kosma et al., 2010; Klee and Giovannoni, 2011). On the contrary, tomatoes with the LSL phenotype differ from the latter mutants in that fruit ripening is not badly impaired, but rather exhibit normal climateric ripening.

The LSL phenotype has been related to the *alc* mutation (e.g., Kopeliovitch et al., 1981; Lobo et al., 1984; Mutschler, 1984a; Casals et al., 2012; Conesa et al., 2014), described in the ‘Alcobaça’ landrace (Almeida, 1961; Leal and Tabim, 1974). First reports for shelf-life in ‘Alcobaça’ were up to 316 days (Leal and Tabim, 1974), although further reports are considerably shorter (up to 33 days; Kopeliovitch et al., 1980; Mutschler 1984b; Mutschler et al., 1988; Mutschler et al., 1992; Dias et al., 2003). The *alc* mutation is present in ‘de Penjar’ from Catalonia and Valencia and ‘de Ramellet’ from the Balearic Islands (Casals et al., 2012; Bota et al., 2014). The ‘delayed fruit deterioration’ mutant (*dfd*) was described as a regionalized cultivar grown in specific areas around the Mediterranean, with dramatically delayed softening and unknown genetic background (Saladie et al., 2007). Such description fits with LSL landraces, while fruit size, shape and shelf-life are similar to ‘de Penjar’ (Rose, unpubl., *cf*. Conesa et al., 2014). Actually, the *dfd* mutant seems to bear the *alc* mutation (Vrebalov et al., 2004; Bota et al., 2014). The *alc* mutation has been suggested as responsible for the LSL phenotype also in ‘da Serbo’ Italian landraces (Mercati et al., 2015). However, its presence has not been reported in Italian landraces, to our knowledge; whereas it is absent in ‘Corbarino’ and ‘Lucariello’ (Tranchida-Lombardo et al., 2018a). This suggests that mutations other than *alc* may also result in the LSL phenotype, particularly in Italian landraces.

Several studies support that *alc* and *nor* mutations are allelic (Tigchelaar et al., 1976; Lobo et al., 1984; Benites et al., 2010; Casals et al., 2012; Kumar et al., 2018). Recently, studies of NAC-family genes described diverse *nor* alleles and *nor*-like genes involved in the tomato ripening process (Wang et al., 2018). Also, Kumar et al. (2018) described two different cDNA products for *nor* added up to *alc* in ‘de Penjar’/‘de Colgar.’ The accessions considered included two from Catalonia (i.e, ‘de Penjar’), one from Málaga and one from Cáceres (i.e., ‘Alcobaça’), (pers. comm. R. Fernández-Muñoz). All bear the *alc* mutation except the latter, from which the two discordant *nor* products were described (Kumar et al., 2018).

The impact of the *alc* mutation on the extended fruit shelf-life was assessed by introducing the mutation in ‘M82’ tomato by CRISPR/Cas9 transformation (Yu et al., 2017). The shelf-life reported was only up to 40 days, which is similar to early reports for ‘Alcobaça,’ and much shorter than that in most LSL landraces. This agrees with past indications that *alc* may be necessary but not sufficient to explain the LSL phenotype in the Mediterranean landraces (Conesa et al., 2014; Casals et al., 2015). Altogether, some controversy exists among the possible existence of different mutations in the MADS-box, SBP-box and NAC-family genes (Seymour et al., 2013; Kumar et al., 2018), which may be responsible for part of the variation reported in shelf-life across LSL landraces.

### Factors Determining the LSL Phenotype

Contrary to non-ripening mutants, most of which have an impairment related to ethylene insensitivity causing deficient ripening and pleiotropic effects on fruit quality (Barry and Giovannoni, 2007; Giovannoni, 2007), ripening in LSL landraces is fully achieved on the vine, and fruit quality is one of the most appreciated traits (Saladie et al., 2007; Siracusa et al., 2013; Bota et al., 2014; Casals et al., 2015; Patanè et al., 2017). Ethylene production and respiration rates in LSL fruits has been poorly studied to date. Saladie et al. (2007) found a delay of seven days to each red-ripe stage in *dfd* as compared to ‘Ailsa Craig’, although the peak in ethylene production was similar, and the climacteric respiratory burst was more pronounced in *dfd* (i.e., ca. 16 *vs*. 12 nl ethylene g^−1^ h^−1^, and 55 *vs*. 25 ml CO_2_ g^−1^ h^−1^). Nevertheless, authors attributed such differences to already known variation occurring within the tomato crop (Saladie et al., 2007).

In a recent screening for genetic diversity across a collection of Italian LSL landraces, Tranchida-Lombardo et al. (2018b) found high variation in ethylene-responsive genes, relating it to the extended fruit shelf-life. However, they found no polymorphism in *nor* and *alc* mutations, suggesting that genetic determinants of the LSL fruit phenotype may be different between Italian and ‘de Ramellet’–‘de Penjar’ landraces. In the ‘Alcobaça’ landrace, it was described an effect of the *alc* mutation related to reduced ethylene production (i.e., 25% less than ‘Rutgers’ cultivar; Mutschler, 1984b). However, postharvest storage attributed to this landrace is commonly lower than 60 days, which differs from the 6–8 months reported for most LSL landraces. This also endorses previous indications that the *alc* mutation may be needed but not sufficient to explain extended fruit shelf-life in LSL landraces (Conesa et al., 2014; Casals et al., 2015). Moreover, this denotes opposite responses attributable to the *alc* mutation on the ethylene production, either reducing it (Mutschler, 1984b) and increasing it (Saladie et al., 2007).

In this regard, Kumar et al. (2018) compared ethylene emission in ‘Ailsa Craig’ with four different ‘de Penjar’ accessions, and found reduced ethylene production in three of the ‘de Penjar,’ but increased in the Penjar-2 accession, emitting slightly higher ethylene at breaker stage than ‘Ailsa Craig’. This suggests that the above indicated opposite responses of *alc*-bearing accessions towards ethylene emission may not be only related to this mutation, but could respond to intrinsic variation within LSL landraces. In fact, Kumar et al. (2018) described novel *NOR* mutations within the studied ‘de Penjar’ accessions, all in the Penjar-1 accession and thus, not related to the differences reported for ethylene production. Further, they found good correlation of ethylene emission with other ripening-related metabolites like jasmonic acid in all the ‘de Penjar’ accessions except Penjar-4. Consequently, results suggest that the LSL fruit phenotype may be also related to non-ethylene mediated ripening regulation, as described from non-ripening mutants (e.g., Moore et al., 2002; Vrebalov et al., 2002). This variability in ethylene production within LSL landraces, uncertainties on the different role of ethylene in LSL *vs*. non-LSL landraces, and the impact of ethylene in fruit quality traits and aroma highlights the importance of deepening in the knowledge of ethylene production pathways within LSL landraces to better understand extended shelf-life and ripening process in tomato.

Apart from ethylene, the maintenance of fully-ripen fruits sound over months of storage at ambient temperature suggests a dramatic slow-down of the respiratory metabolism and, particularly, an effective prevention of transpirational water loss in LSL fruits (Bargel and Neinhuis, 2004; Saladie et al., 2007; Conesa et al., 2014; Casals et al., 2015). A main effect of fruit cuticle properties on non-LSL tomato shelf-life has already been suggested (Bargel and Neinhuis, 2004; Bargel and Neinhuis, 2005; Domínguez et al., 2011), and related to increased waxes during ripening in *nor*, *rin* and ‘Alcobaça’ (Kosma et al., 2010). Increased cutin accumulation, but not cuticle thickness, seems important in *dfd* (Saladie et al., 2007). Therefore, it seems that cuticle properties –composition and cutin matrix architecture—in fruits with the LSL phenotype may be key in maintaining fruit impermeability.

Studies in tomato ripening mutants suggest a role for fruit water loss in triggering ethylene synthesis leading to ripening finalization and over-ripening. This agrees with initiation of fruit ripening in the tomato wild relatives *Solanum chilense* and *S. peruvianum,* only once fruit is detached from the plant (reviewed in Barry and Giovannoni, 2007). Consequently, the ability to maintain fruit water for longer time can also be a factor preventing over-ripening in LSL fruits.

Diverse authors suggested the thick skin and high antioxidant content as responsible for the extended fruit shelf-life in Italian landraces (Barbagallo et al., 2008; Siracusa et al., 2012; Mercati et al., 2015; Patanè et al., 2016; Manzo et al., 2018; Renna et al., 2018). Moreover, fruit size has been inversely correlated with shelf-life in ‘Alcobaça’ (Leal and Tabim, 1974; Mutschler et al., 1992) and ‘de Penjar’ (Casals et al., 2012). This correlation was non-significant in ‘de Ramellet,’ probably because of variation in shelf-life sensitivity to water availability (Conesa et al., 2014) and high variation in fruit size and shape (Bota et al., 2014). In fact, many Italian LSL landraces have smaller fruits than ‘de Ramellet’ and ‘de Penjar’ (< 21 g; Siracusa et al., 2012; Patanè et al., 2016) but the shelf-life is not longer than that reported for the latter landraces, being two- to ten-fold bigger (Casals et al., 2012; Bota et al., 2014).

Despite the huge variation in shelf-life reported for LSL tomatoes (e.g., Leal and Tabim, 1974; Mutschler et al., 1988; Saladie et al., 2007; Casals et al., 2011; Conesa et al., 2014; Manzo et al., 2018; Tranchida-Lombardo et al., 2018a) could result from different mutations and even epigenetics (Mirouze and Paszkowski, 2011; Osorio et al., 2013; Schmitz et al., 2013; Zhong et al., 2013; Bressan et al., 2014; Van Oosten et al., 2014; Giovannoni et al., 2017), factors affecing shelf-life in LSL fruits like fruit size (Leal and Tabim, 1974; Mutschler et al., 1992; Casals et al., 2011; Bota et al., 2014), titratable acidity (Bota et al., 2014), sugar content (Kumar et al., 2018), antioxidant content (Barbagallo et al., 2008; Siracusa et al., 2012; Mercati et al., 2015; Patanè et al., 2016; Renna et al., 2018) are to a high extent influenced by cultivation conditions. Therefore, a part of the reported variation may be environmentally-driven.

In fact, fruit shelf-life in ‘de Ramellet’ showed some interaction with water availability during cultivation, denoting that traits conferring the LSL phenotype were impaired by too high irrigation in some landraces, but not others (Conesa et al., 2014). Since ‘de Ramellet’ is homozygous for *alc*, this mutation is not sufficient to explain the variation in shelf-life within this landrace. Hence, too high irrigation could be impairing cuticle properties in the sensitive genotypes, either composition, component proportions or biomechanic properties, resulting in impaired LSL phenotype (Conesa et al., 2014; Conesa et al., 2015).

## Drought Tolerance in Mediterranean LSL Landraces

In agricultural systems, drought refers to a scenario where water transpired by the plant cannot be fully replaced by soil water availability, forcing plants to reduce transpiration by stomatal closure, which limits growth and yield (Tardieu et al., 2018). Drought tolerant crops have mechanisms allowing plants to withstand eventual or permanent water shortage with lower yield reduction as compared to non-tolerant crops (Tuberosa, 2012). These mechanisms may be found at very different morphological, physiological and molecular levels, as evidenced by a huge amount of studies in diverse crops (reviewed in Tuberosa, 2012; Nuccio et al., 2018; Sreeman et al., 2018 and Tardieu et al., 2018). Particularly in Mediterranean LSL tomato landraces, studies of drought tolerance have focused mainly on leaf gas exchange traits, water use efficiency, solute accumulation and hormonal signalling, which biases this review. Hence, this also highlights the need to explore further mechanisms improving drought tolerance in this tomato landrace group.

**Figure 3:**
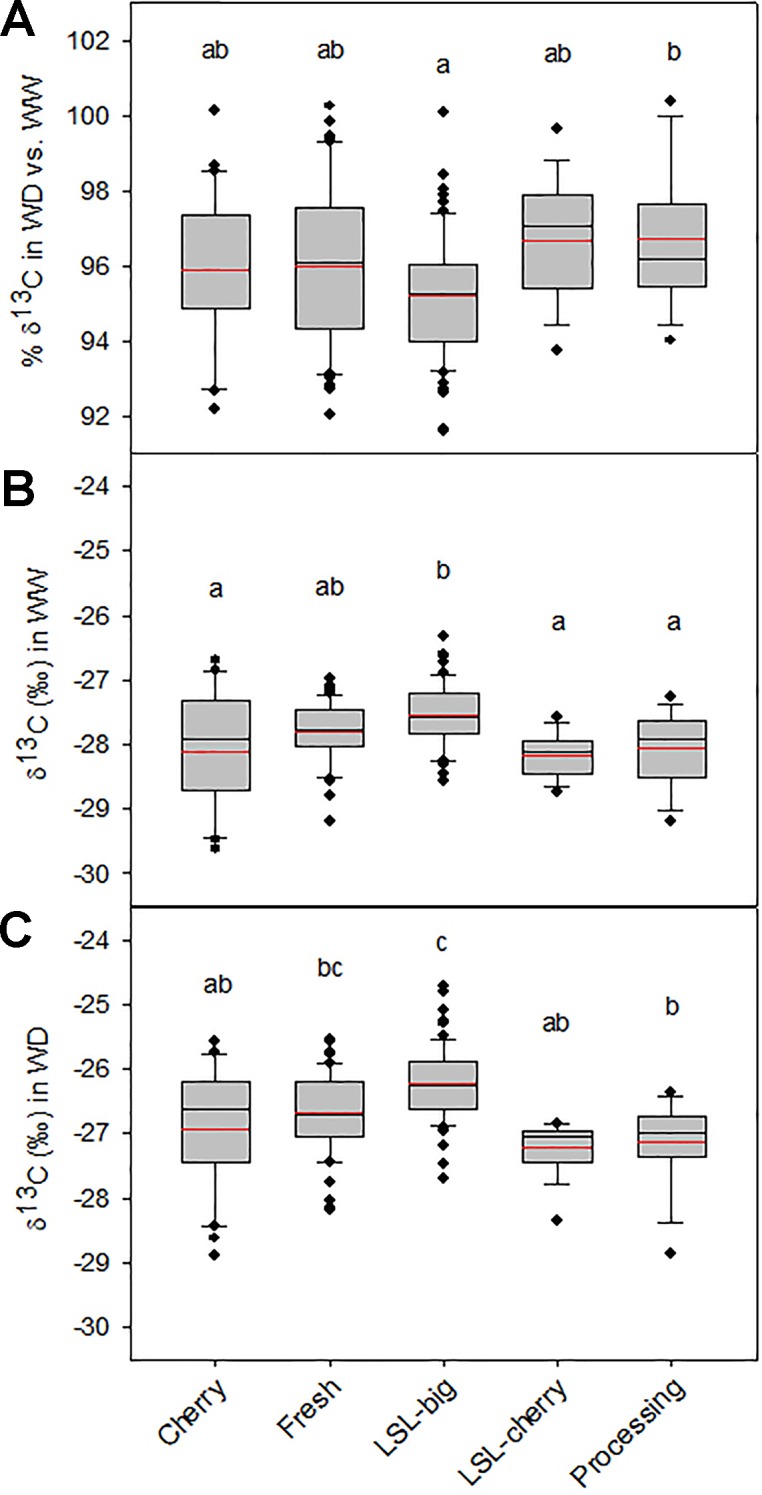
Water use efficiency as inferred from δ^13^C (‰) isotopic composition of leaves from tomato plants grown under full irrigation (WW) and water deficit (WD) treatments in Fullana-Pericàs et al. (2019). Tomato accessions (n = 171) were separated in five groups depending on the accession type, including non-long shelf-life cherry (Cherry; n = 29), fresh market (Fresh; n = 50), long shelf-life from eastern Iberian Peninsula and Balearic Islands (LSL-big; n = 63) and from Italy (LSL-cherry;n = 16), and processing accessions (Processing; n = 13). **(A)** Percent of δ^13^C in WD as compared to WW. ANOVA differences between WD and WW were significant in all groups (P < 0.001). **(B**) Values of δ^13^C in WW and **(C)** in WD. For each accession group, boxplots represent the average and the median (red and black lines inbox, respectively), the 75% interval (box), the 90% interval (error bars) and the outliers (isolated points). In each plot, letters on top indicate ANOVA-Tukey differences among groups (P < 0.05).

Carbon isotopic composition (δ^13^C) in leaf tissue has proven to be an efficient and reliable method to assess water use efficiency in plants (e.g., Farquhar et al., 1982; Condon et al., 1990; Donovan and Ehleringer, 1994; Bacon, 2004; Galmés et al., 2011). It was measured in a diverse array of traditional tomato accessions including cherry, fresh market, processing and LSL (Fullana-Pericàs et al., 2019), grown under full irrigation (WW) and water deficit (WD). The LSL group included accessions from Eastern Iberian Peninsula and Balearic Islands (LSL-big) and Italian accessions (LSL-cherry). δ^13^C increased in all groups under WD as compared to WW (**Figure 3**) showing that, irrespective of the tomato type, traditional accessions have mechanisms allowing adaptation to water shortage through increased water use efficiency.

When comparing among groups, LSL-big had highest (less negative) δ^13^C under both water treatments (**Figures 3B**, **C**), which would indicate lower water use efficiency. Higher δ^13^C frequently occurs under drought and is related to stomatal closure to prevent water loss. However, low δ^13^C values found also under WW denote that this is an intrinsic trait in LSL-big, which could relate to constitutive tighter stomatal control as compared to the remaining groups, and result in higher improvement of water use efficiency.

It is noteworthy that in both treatments the largest difference in δ^13^C among groups was between LSL-big and LSL-cherry, highlighting strikingly different strategies to adapt to harsh conditions between both LSL groups. Contrary to LSL-big, the lowest δ^13^C values in LSL-cherry, especially under WD (**Figures 3B**, **C**), pointed to an adaptive strategy minimizing operation with partially closed stomata, either through deeper roots providing higher water availability, or with a water-conservative stomatal behaviour (i.e., fast closure under drought). The big plant size and high vigor and yield in the LSL-cherry under drought (Andreakis et al., 2004; Sinesio et al., 2007; Lisanti et al., 2008) better points to the former strategy. The lowest difference between treatments in δ^13^C occurring in LSL-cherry (**Figure 3**) also endorsed a drought-tolerance strategy involving mechanisms to increase access to water. In this regard, LSL-big had the highest difference between treatments in δ^13^C (**Figure 3**), denoting the existence of further mechanisms increasing drought tolerance in this group.

As compared to WW, stomatal closure under WD to prevent water loss is evidenced in a reduction in stomatal conductance (*g*_s_), meaning either lower water loss and lower CO_2_ intake through the stomata. Consequently, net photosynthesis (*A*_N_) is also reduced. Adaptive mechanisms at leaf level allow plants to minimize the reduction in *A*_N_ as compared to *g*_s_, that is, increase the intrinsic water use efficiency (*WUE*i, as *A*_N_/*g*_s_). Leaf adaptations to increase *WUE*i in ‘de Ramellet’ occur at leaf surface and leaf mesophyll levels. At leaf surface level, stomatal anatomy modifications expected to provide higher *WUE*i are related to smaller stomata, higher stomatal density, and higher abaxial/adaxial ratio (Franks and Beerling, 2009). In agreement with that, ‘de Ramellet’ showed reduced stomatal size and increased stomatal density under WD as compared to WW. However, it is remarkable that WD adaptation resulted in increased stomatal density because of an increased adaxial density (Galmés et al., 2013). The lack of similar studies in other LSL, and even non-LSL landraces limits understanding if this is a common strategy for drought adaptation in Mediterranean landraces.

At leaf mesophyll level, mechanisms to minimize growth and yield impairment under drought due to lower CO_2_ intake through stomata involve improvement of the CO_2_ delivery pathways from the substomatal cavity to the Rubisco, leading to improved mesophyll conductance (*g*_m_). This is a frequently described mechanism across plant groups and growth forms (Flexas et al., 2013a). Gas exchange and leaf anatomy measurements in ‘de Ramellet’ unravelled the mechanistic basis of improved *g*_m_ under water deficit conditions in this LSL landrace (Galmés et al., 2011; Galmés et al., 2013). In ‘de Ramellet,’ yield correlated positively with *A*_N_ and negatively with *WUE*i, indicating that yield increases were at expenses of proportionally higher increases in *g*_s_ (i.e, water consumption), (Galmés et al., 2011). As compared to WW, there was an increase of *WUE*i under WD, achieved through increased g_m_ per unit water transpired, i.e., increased *g*_m_/*g*_s_ ratio (Galmés et al., 2013). Despite stomatal closure under WD resulted in reduction of both *g*_s_ and *g*_m_, the differential degree of reduction was explained by leaf anatomical adaptations occurring under WD.

**Figure 4:**
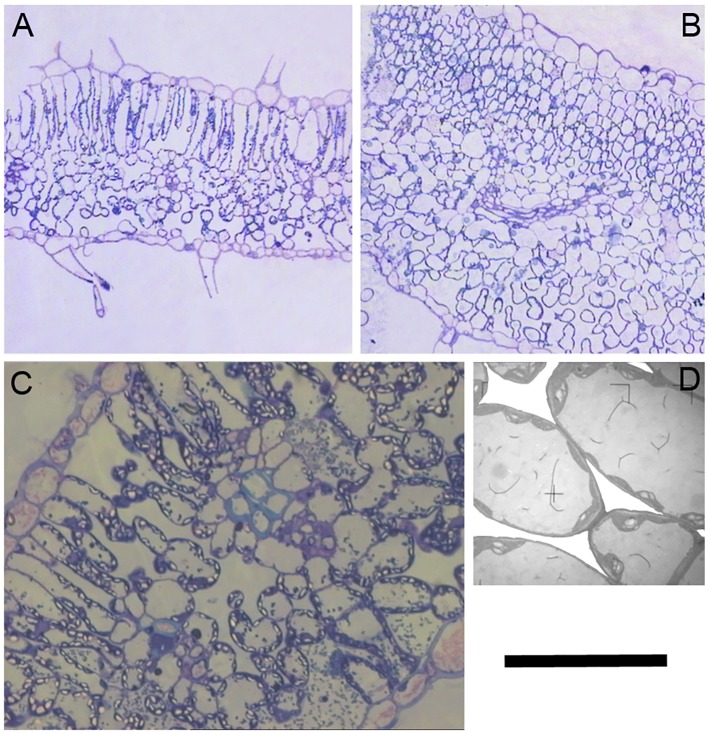
Cross sections of a ‘de Ramellet’ leaflet formed under **(A)** well-watered and **(B)** water stress (WS) conditions. **(C)** Detail of the mesophyll cells and airspaces under WS. Notice the chloroplasts (bright) tightly positioned against the cell membrane and cell wall. **(D)** Magnification of a few cells in C to highlight intercellular airspaces, that have been whitened to ease visualization. Black bar represents 200 µm in A and B, 40 µm in C and 10 µm in D. All images from the experiment described in Galmés et al. (2013).

**Figure 5:**
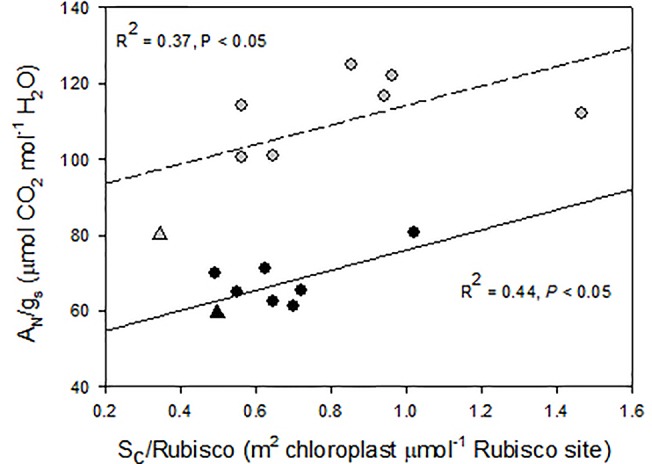
Relationship between intrinsic water use efficiency (net photosynthesis and stomatal conductance ratio, A_N_/g_s_) and the ratio between the mesophyll cell surface exposed to airspaces (S_c_) and the Rubisco concentration. Dots are different “de Ramellet” accessions grown under well-watered (black, soild regression line) and water-stress (grey, dashed regression line) conditions. The triangles represent a processing accession used as control. Plotted from data in Galmés et al. (2013).

A close inspection of mesophyll anatomy showed a notorious increase in intercellular airspaces (**Figures 4A**, **B**), resulting in increased surface of cells having chloroplasts exposed to airspaces (*S*_c_) under WD, thus windows for chloroplasts to access CO_2_. Further, chloroplasts were tightly positioned against the cell membrane to minimize the CO_2_ pathway (**Figures 4C**, **D**; Galmés et al., 2013). The positive correlation between *g*_m_ and S_c_ under WD demonstrated that *WUE*i improvement was a consequence of increased *g*_m_ (as compared to *g*_s_) achieved through increased *S*_c_. Since CO_2_ is finally fixed by Rubisco, the *S*_c_/[Rubisco] ratio may be a key trait explaining the efficiency in CO_2_ fixation (Onoda et al., 2017). Accordingly, this ratio positively correlated with *WUE*i in both water treatments, although for the same *S*_c_/[Rubisco] the *WUE*i was scaled-down under WW as compared to WD (**Figure 5**). In this regard, the non-‘de Ramellet’ accession was the single one reducing the *S*_c_/[Rubisco] ratio under WD (**Figure 5**) and thus, the described leaf anatomical adaptation in ‘de Ramellet’ is not occurring in all tomato varieties. Further knowledge on stomatal and mesophyll characteristics in Mediterranean landraces, and their response to drought conditions, may unravel important patterns suitable to breed for novel tomato cultivars more tolerant to water scarcity.

Diverse drought experiments including landraces from Sicily and Campania denoted the lack of *A*_N_ and *g*_s_ differences among accessions, irrespective of the severity of the stress (Patanè et al., 2016; Guida et al., 2017; Giorio et al., 2018). Moreover, *A*_N_, *g*_s_ and *WUE*i values under drought were close to those reported for ‘de Ramellet’ (Galmés et al., 2011) and, similarly, the *A*_N_ to *g*_s_ relationship indicated a saturation of *A*_N_ and thus, a disproportionate, suboptimal water consumption under non-stressing conditions (Guida et al., 2017; Giorio et al., 2018). Overall, despite maximum *A*_N_ and *g*_s_ varied considerably among Mediterranean LSL landraces and studies, *WUE*i under stress was grossly similar across landraces, with values at low *g*_s_ (i.e., 0.15–0.30 mol m^−2^ s^−1^) close to 90–110 µmol mol^−1^ in “de Ramellet” (Galmés et al., 2011), Sicilian landraces (Guida et al., 2017; Giorio et al., 2018) and “Vesuviano” (Guida et al., 2017).

In diverse Sicilian LSL landraces, Patanè et al. (2016) found that accessions with highest yield had also among the highest *WUE*i, standing out very tolerant and productive landraces like ‘Locale di Custonacci’. In such LSL landraces, drought tolerance was linked to leaf proline accumulation. Since proline accumulation is related to osmoregulation capacity, this could agree with δ^13^C results above (**Figure 3**) suggesting that the drought tolerance strategy in LSL-cherry accessions is better related to maintenance of water intake than to water conservation. Nevertheless, important differences in the response to drought have been described in Italian landraces. In ‘Locale di Salina 6’ and ‘Pizzutello di Sciacca,’ Giorio et al. (2018) found a trade-off between *g*_s_ and ABA accumulation. In ‘Crovarese’ under short-term water stress, Tamburino et al. (2017) also found high reduction of *g*_s_ and increase of ABA and proline. In several LSL landraces from Campania including ‘Lucariello,’ ‘Crovarese’ and ‘Giallo Benaventano,’ Landi et al. (2017) described the constitutive activation of ROS detoxification machinery as a fast response under stress. This involved enzymes that reduced the accumulation of ROS resulting from drought-induced photorespiration, with a parallel proline accumulation, thus also triggering osmotic adjustments as a response to drought. Deepening in the role of proline and ROS detoxification in Mediterranean LSL landraces, and the comparison of the drought response in LSL landraces from different regions may unmask alternative strategies to the described above for ‘de Ramellet’ to adapt to water scarcity.

Leaf veins are a necessary connection for water supply to the stomata and thus, a key link between leaf function and stomatal and mesophyll anatomy (Brodribb and Holbrook, 2003; Brodribb et al., 2010; Scoffoni et al., 2016). This link has been described across diverse species and growth forms (e.g., Brodribb et al., 2007; Flexas et al., 2013b; Sack et al., 2015), but has been much less explored within crop species. In a comparison of domesticated tomato accessions including ‘de Ramellet’ with the tomato wild relative species (*Solanum* sect. *Lycopersicon*, sect. *Lycopersicoides* and sect. *Juglandifolia*), the domesticated had consistently larger leaf size and higher *A*_N_. This was achieved with the lowest vein density and more elastic cell walls (i.e., lowest bulk modulus of elasticity, ϵ_max_), (Conesa et al., unpubl.). Thus, as compared to wild tomato species, the domesticated accessions minimized the space occupied by the vein system, which maximizes the space occupied by mesophyll cells, maximizing *A*_N_. This contravenes general patterns in plants linking high *A*_N_ with high vein density and high hydraulic conductance (e.g., Brodribb et al., 2007; Sack et al., 2015), highlighting that crop domestication might have led to alternative trait relationships in particular species like tomato. Nevertheless, within ‘de Ramellet’ higher vein density was related to higher hydraulic conductance (Galmés et al., 2013). Knowledge on the impact of leaf venation and hydraulics modifications under drought across diverse tomato LSL landraces may highlight important traits and trait-relationships at leaf level endowing superior capacity to overcome drought, suitable to be considered in breeding programs aimed to adapt tomato crop to future climate change conditions.

## Trait Variability Within Mediterranean LSL Landraces

A common characteristic in landraces is the high heterogeneity in most plant and fruit traits, resulting from variable selection criteria across local populations, due to the close link between landraces and local population heritage (Zeven, 1998; Camacho-Villa et al., 2005). This is particularly true for in the Mediterranean LSL landraces (Bota et al., 2014; Patanè et al., 2016; Siracusa et al., 2018), making the high trait variability a characteristic trait defining many of such landraces.

### Variation in Fruit Morphology

There is a notorious difference in fruit shape between LSL landraces from Italy and from the Iberian Peninsula and Balearic Islands. The latter are in general round to flat fruits, while Italian landraces are usually elongated, oval, pear-shaped and frequently bear a stylar end tip. Differences exist also in fruit size, with most Italian LSL landraces having small fruits, up to 30–40 g, while most Iberian and Balearic LSL landraces have bigger fruits (**Table 2**). To our knowledge, variation in fruit size and shape has been widely characterized only in the Balearic ‘de Ramellet’ (**Figure 1**), which is one of the most variable Mediterranean LSL landraces. Most fruits are flat or round, existing also accessions with ellipsoid, heart, obovoid, oxheart, and rectangular fruit shapes (**Table 3**; **Figure 1**). Despite rare, up to 4% of the accessions had some degree of stylar end tip, all being elongated and pear-shaped fruits (**Figure 1**). Since the frequency of this trait in Italian tomatoes, this could represent some degree of introgression between Mallorcan and Italian landraces (Conesa et al., 2010). Added to the variation in fruit shape and size, fruit colour is also variable within the Balearic ‘de Ramellet.’ While most are kind of ‘pink tomatoes’ (with a colourless cuticle lacking flavonoids), red fruited landraces (with yellow cuticle) also occur in ‘de Ramellet’ (Conesa et al., 2015). Most landraces have yellow to orange coloration in the half part closest to the pedicel (**Figures 2A**, **B**), which is attributable to pleiotropic effects of the *alc* mutation. This coloration-deficient trait is appreciated by local consumers and helps recognizing ‘de Ramellet’ from derived F_1_ hybrids, with consistently uniform coloration.

**Table 3:** Descriptive plant and fruit traits in “de Ramellet” (158 accessions) cultivated outdoors in Mediterranean summer under low irrigation (WW; see **Table 2** for details).

Fruit shape	% acc.	Fruit weight	% acc.	6–month shelf–life	% acc.
Heart	4.9	<25 g	7.6	100%	2.1
Ellipsoid	6.3	25–50 g	50	99–75%	17.6
Flat	56.3	50–70 g	38	74–50%	38
Round	30.3	>70 g	4.4	49–25%	24.6
Obovoid	1.4			24–1%	13.4
Oxheart	0.7			0%	4.2
Rectangular	0.7				
**Plant volume**	% acc.	**Plant habit**	% acc.	**Num. branches**	% acc.
<100 dm^3^	7	Climbing/erect	47	3–4	17
100–200 dm^3^	25	Typical indet.	43	5–6	32
200–400 dm^3^	50	Creeping	8	7–8	32
400–500 dm^3^	10	Small bush	2	9–10	6
>500 dm^3^	8			11	3

Data shown represents the % of accessions in each category or interval. Fruit traits include shape categories based on Tomato Analyzer (Brewer et al., 2006), average fruit weight, and accessions with >50% of fruits intact after 6–month shelf–life (170 days). Plant morphological traits are plant volume (based on two horizontal axes and maximum height), plant habit, and the number of principal branches. Data from, or recalculated from, Ochogavía et al. (2011) and Bota et al. (2014).

### Variation in Plant Morphology, Agronomic Traits and Yield

Similar to fruit morphology variation, plant traits are also diverse within ‘de Ramellet’ (Ochogavía et al., 2011; Bota et al., 2014). When grown with no tutoring and no pruning, most accessions had plant volumes of 200–400 dm^3^ (**Table 3**). Regarding plant habit, most plants were climbing-erect, with 5–8 principal branches, and 8% of the accessions had potato leaf instead of the typical 5–9 leaflets leaves. There was also variation in phenology, with most accessions having relatively late production as compared to non-‘de Ramellet,’ and on average most productive period ranged between 85–120 days after transplantation (Ochogavía et al., 2011). Although root traits have not been assessed in detail in ‘de Ramellet,’ the variability in aerial plant parts' morphology suggest also differences in root, either extension and growth patterns.

Agronomic evaluation of 48 ‘de Ramellet’ accessions grown outdoors under well-watered (WW) and water deficit (WD) treatments showed a low effect of irrigation and field conditions on fruit size (**Table 4**; Fullana-Pericàs et al., 2019). Most accessions produced 1.7–4.0 kg plant^−1^, and very variable fruit number per plant, from 25–150. In ca. 30% of the accessions, yield was higher under WD than under WW or invariable between treatments, denoting a low impact of water shortage, or a negative effect of higher water availability on yield. Results are similar in ‘de Penjar,’ being also variable among accessions and experiments (**Table 2**).

**Table 4:** Agronomic traits in a selection of 48 ‘de Ramellet’ accessions cultivated outdoors in Mediterranean summer under low irrigation (WW) and water deficit (WD; see **Table 2** for details), at a commercial production field.

Yield per plant (total)	WW (% accessions)	WD (% accessions)
<600g	8.3	8.3
600–1,150g	4.2	10.4
1,150–1,700g	8.3	22.9
1,700–2,250g	16.7	16.7
2,250–2,850g	27.1	22.9
2,850–4,000g	22.9	16.7
4,000–6,000g	12.5	2.1
Average yield (g plant–^1^)	2603.4 ± 178.7	2017.1 ± 145.4
Average % WD vs WW	82.4 ± 4.3 (10–157%)	
% acc. WD > WW	29.2% (539–3648 g)	
**Num. fruits per plant (total)**	**WW (% accessions)**	**WD (% accessions)**
<25	2.1	10.4
25–50	18.8	18.8
50–75	10.4	22.9
75–100	29.2	22.9
100–150	29.2	22.9
150–200	8.3	0.0
>200	2.1	2.1
Average num. fruits (plant^−1^)	90.2 ± 6.1	73.6 ± 5.7
Average % WD vs WW	85.5 ± 4.6 (15–168%)	
% acc. WD > WW	33.3% (48–235)	
**Fruit weight (marketable)**	**WW (% accessions)**	**WD (% accessions)**
<25g	4.2	6.3
25–50g	22.9	20.8
50–70g	56.3	56.3
>70g	16.7	16.7
Average fruit weight (g)	57.4 ± 2.0	54.6 ± 2.4
Average % WD vs WW	101.1 ± 6.0 (31–340%)	
% acc. WD > WW	35.4% (43–89 g)	

The accession selection represents the highest genetic diversity in the UIB–collection. Yield and fruit number are per plant and correspond to total production (i.e., marketable and damaged), while average fruit weight corresponds only to marketable fruits. In each interval, values correspond to the % of accessions in the WW and WD treatments. The global effect of WD on each trait is indicated as the average and SE in each treatment (asterisk in WD indicates significant differences between treatments; P < 0.05), the average of the % in WS as compared to WW (the variation range shown in brackets), and the % of accessions with higher value in WS than in WW (the variation range of such accessions under WD shown in brackets). Data recalculated from Fullana–Pericàs et al. (2019).

Literature reports for yield in Italian LSL landraces also denote large differences across landraces and experiments, with values in open-field and rain-fed conditions ranging from 12–16 t/Ha in e.g. ‘Locale di Salina 6,’ ‘Locale Giallo di Basilicò,’ ‘Pizzutello di Sciacca’ and the commercial ‘Principe Borghese,’ to ca. 100 t/Ha in ‘Corbarino’ (**Table 2**). Yield values per plant reported for ‘Piennolo Vesuviano’ (ca. 5.2 kg plant^−1^) and ‘Locale di Salina 6’ (ca. 3.2 kg plant^−1^) are not very different to those in ‘de Ramellet’ and ‘de Penjar,’ although fruit number per plant is dramatically higher in Italian landraces, and often associated to the smaller fruit size (**Table 2**).

### Variation in Fruit Quality Attributes

Despite selection for the LSL phenotype converged in the need for fresh vegetables over-winter, organoleptic and physical properties of the fruit is contrastingly different across regions (Causse et al., 2010). In the Balearic Islands, ‘de Ramellet’ is recognized by higher acidity than most tomatoes (> 1 g citric acid 100 ml^−1^ at open-field; **Table 2**), whereas most Italian LSL landraces have lower values (ca. 0.2–0.4 g citric acid 100 ml^−1^ at open-field; **Table 2**). On the contrary, sugar content is lower in ‘de Ramellet’ (ca. 6.0°Brix) than in most Italian landraces (ca. 7-8 °Brix), (**Table 2**), indicating that sugar content has been an important selective trait in Italian LSL landraces. Reports of acidity and sugar content for ‘de Penjar’ are similar to ‘de Ramellet’ (**Table 2**).

The variation in fruit quality traits among LSL landraces, and among reports for the same landrace, may be in part the result of plants grown under different cultivation practices and environmental conditions, having important effects on fruit quality (e.g., Kirda et al., 2004; Patanè and Cosentino, 2010; Casa and Rouphael, 2014; D'Esposito et al., 2017). Cultivation conditions rendering smaller fruits, like drought stress, are in general resulting in a ‘concentration’ effect and ‘tastier’ fruits (Zanor et al., 2009; Panthee et al., 2013; Figàs et al., 2015; Tieman et al., 2017). Fullana-Pericàs et al. (2019) compared fruit quality attributes in diverse ‘de Ramellet’ grown under WW and WD treatments. WD significantly increased sugar content, although acidity differences between WW and WD were non-significant. Nevertheless, acidity reports for ‘de Ramellet’ in further experiments have been contrastingly different (**Table 2**), which indicates an effect of the environment and agronomical practices irrespective of, or in combination with, water availability. As compared to rain-fed conditions, the lower sugar content under irrigation has also been reported in Italian landraces like ‘Locale di Salina 6,’ ‘Piennolo Vesuviano’ and ‘Corbarino,’ (**Table 2**).

On the one hand, the variation in fruit quality traits among LSL landraces denotes that the LSL phenotype is mostly unlinked to fruit quality attributes, agreeing with the different taste and uses across Mediterranean landraces. On the other hand, this describes a diverse array of quality attributes and fruit morphologies in the LSL landraces, making those a very rich resource to improve flavour linked to extended shelf-life.

There is also variation in fruit quality traits in the LSL landraces during postharvest conservation, with decreased sugar content and titratable acidity, and increased pH and firmness, over six-month shelf-life in ‘de Ramellet’ (**Table 5**). Very similar results have been reported for ‘de Penjar,’ with a general decrease of different sugars and volatile compounds during conservation, although the profile for acids was variable (Casals et al., 2015). In both ‘de Ramellet’ and ‘de Penjar,’ most changes occurred during the first two months, with lower changes afterwards. Trends for firmness showed variable patterns in ‘de Ramellet,’ with a dramatically increase during the first two months, followed by a slow decrease afterwards, globally leading to increased firmness after six months (Ochogavía et al., 2011; Bota et al., 2014).

**Table 5:** Fruit quality parameters corresponding to ‘de Ramellet’ accessions in **Table 3**.

	Harvest	Max	Min	2 months	6 months
Firmness (kg cm^−2^)	1.14 ± 0.04a	1.82–2.86	0.19–0.60	1.80 ± 0.03c	1.57 ± 0.04b
Sugar content (°Brix)	5.90 ± 0.06c	7.13–8.75	4.00–5.00	5.48 ± 0.07b	5.10 ± 0.08a
Titratable acidity (g citric ac. 100 ml^−1^)	1.48 ± 0.03c	1.95–2.75	0.80–1.00	1.09 ± 0.02b	0.71 ± 0.02a
pH	3.85 ± 0.01a	4.11^−^4.28	3.27^−^3.57	4.19 ± 0.01b	4.49 ± 0.02c

Fruit firmness (with penetrometer), sugar content (total soluble solids), titratable acidity and pH are shown at harvest and after two and six months (55 and 170 days, respectively) of postharvest storage in a ventilated shed at ambient temperature, as frequently performed by self-consumption growers. Values correspond to averages and standard error for all the ‘de Ramellet’ accessions tested. Letters denote statistically significant differences within each parameter by ANOVA-Tukey (P < 0.001). At harvest time, variation ranges of the 10 accessions with maximum (Max) and with minimum (Min) values are shown to demonstrate variability in the extremes for each trait. Data from Ochogavía et al. (2011) and Bota et al. (2014).

## Mediterranean LSL Landraces as a Resource for Improving Tomato Crop in a Climate Change Scenario

Most breeding efforts in tomato focused earlier in increasing yield and biotic stress resistances (Bai and Lindhout, 2007; Foolad, 2007; Labate and Robertson, 2012; Foolad and Panthee, 2012; Lin et al., 2014). In the 1980-90 decades there was also interest in extending fruit shelf-life, seeking for fruits supporting days-to-weeks export and storage, and having low shipping and transportation damage (Bai and Lindhout, 2007; Foolad, 2007; Díez and Nuez, 2008; Arah et al., 2015). More recently, there has been increasing interest in breeding for drought tolerance, aiming to adapt the tomato crop to climate change conditions, since water scarcity will be a main limitation to yield in important productive areas for tomato like the Mediterranean basin (Lane and Jarvis, 2007; Rockstrom et al., 2007; Lobell and Gourdji, 2012; Dwivedi et al., 2016). However, most of the improvement programs related to extended shelf-life and drought tolerance involved mutants (e.g., *rin*, *nor*; Garg et al., 2008; Klee and Giovannoni, 2011) and wild tomato species (e.g., *Solanum pennellii*; Bolger et al., 2014), respectively.

**Figure 6:**
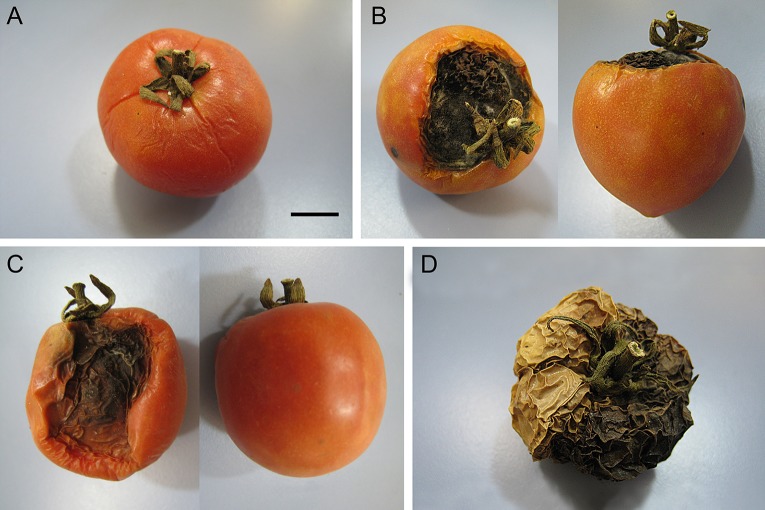
Resistance of ‘de Ramallet’ fruits during post-harvest storage. **(A)** Fruit with slight wrinkling due to water loss six months after harvest. **(B)** Fruit with important scarification a month after a microbial attack, showing the zone of attack (left) and the rest of the fruit (right) remaining intact, without wrinkling. **(C)** Fruit with an important microbial attack emptying half part of the fruit (left) but not the other part (right) which remains intact. Fruits as in B and C can remain as in the picture up to 6 months. **(D)** Fruit completely dry after suffering a microbial attack during postharvest storage. Notice that the dark part was attacked and that the clear half of the fruit maintained integrity until complete water loss. The fruit remains as in the picture for decades with no further deterioration.

Paradoxically, the Mediterranean LSL landraces, with dramatically extended shelf-life, and selected for centuries under severe drought conditions, are an almost neglected genetic resource for tomato improvement. Unlike many non-LSL varieties, LSL landraces do not need to be harvested at breaker stage and finish ripening in chamber-storage to increase shelf-life. Furthermore, due to the ability to maintain fruits sound, LSL tomatoes have higher resistance to microbial attack than most tomatoes, and high cicatrization capacity after damage on the vine like cracking, which probably enhances microbial attack prevention (pers. obs.; **Figure 6**). Thus, the LSL phenotype is a valuable trait to reduce costs of harvesting and storage previous to commercialization (Arah et al., 2015).

Flavour has been largely neglected during tomato improvement and currently many consumers demand tomatoes tasting “like before.” Notorious efforts are being made to decipher determinants of tomato flavour and aroma in order to breed for it in the near future (Brugarolas et al., 2009; Zanor et al., 2009; Causse et al., 2010; Klee and Tieman, 2013; Tieman et al., 2017). Different from biotic and abiotic resistances, for which wild relatives have been a key resource (Foolad, 2007), yearned tomato flavour relies on extant heirloom and landraces (Brugarolas et al., 2009; Figàs et al., 2015; Baldina et al., 2016; Tieman et al., 2017). In this regard, preferences for taste are very dependent on the uses and regions considered and thus, a diverse array of fruit quality traits must be considered (Causse et al., 2010). Particularly in the Mediterranean, the large variation observed for LSL landraces in fruit size, shape and quality attributes, constitute a very suitable resource to seek for ancient tomato flavour, and as a source to breed for improved tomato taste.

Agricultural biodiversity is a prime resource for novel breeds adaptation (Lane and Jarvis, 2007; Frison et al., 2011). The vast majority of LSL landraces are maintained in home-gardens and orchards for self-consumption, through own selection and seed storage, with a tight and ancient link to the local gastronomy and culture (e.g., Andreakis et al., 2004; Siracusa et al., 2012; Bota et al., 2014; Mercati et al., 2015; Patanè et al., 2016). Changes in cultural practices and lower interest in agriculture in upcoming generations leads to a dramatic genetic erosion in landraces across the Mediterranean (Cebolla-Cornejo et al., 2007; Terzopoulos and Bebeli, 2008; Mazzucato et al., 2008; Bota et al., 2014; Cortés-Olmos et al., 2015; Mercati et al., 2015). Consequently, there is urgent need for researchers, breeders, growers, public managers and policy makers to work collaboratively towards the characterisation, conservation, develop protection regulations and revalorisation of Mediterranean LSL landraces, to preserve this rich local heritage (Zeven, 1998; Camacho-Villa et al., 2005; Negri et al., 2009; Frison et al., 2011; Elia and Santamaria, 2013; Corrado et al., 2014; Garcia-Mier et al., 2014; Dwivedi et al., 2016; Casañas et al., 2017).

## Concluding Remarks

The Mediterranean LSL tomatoes are a group of landraces with dramatically extended fruit shelf-life after harvest, mostly occurring in Eastern Iberian Peninsula, the Balearic Islands, and Southern Italy. In these areas, LSL landraces are commercialized in local markets and are an important part of the local culture and heritage. Besides extended shelf-life, most LSL landraces are drought tolerant, as a consequence of ancient selection under Mediterranean summer conditions with poor irrigation or rain-fed. Therefore, LSL landraces constitute an alternative to wild species as a source of genes to improve drought stress tolerance. Moreover, the large variation in fruit morphology, fruit quality traits and flavor within and among landraces makes the LSL landraces an attractive source to breed for future tomato cultivars with a genetic background conferring extended shelf-life and drought tolerance. Currently, genetic erosion is a prime in most Mediterranean LSL landraces and there is an urgent need to preserve and revalue this important genetic resource, which has notorious traits suitable to improve tomato for fruit quality, shelf-life and cultivation under the predicted climate change conditions.

## Data Availability Statement

All datasets generated for this study are included in the article.

## Author Contributions

MC and JG conceived the idea and the review scheme. MC drafted the manuscript. All authors did significant contributions improving the final version of the manuscript.

## Funding

This project has received funding from the European Union's Horizon 2020 research and innovation programme under grant agreement No 727929 (TOMRES), No 634561 (TRADITOM) and No 679796 (TomGEM). Research has been also supported by the Spanish Ministry of Economy and Competitiveness (MINECO) project AGL2013-42364-R (TOMDRO), and the Government of the Balearic Islands grants BIA20/07, BIA07/08, BIA09/12 and AAEE56/2015. MF-P has a pre-doctoral fellowship (FPI/1929/2016) granted by the Government of the Balearic Islands.

## Conflict of Interest

The authors declare that the research was conducted in the absence of any commercial or financial relationships that could be construed as a potential conflict of interest.
